# Association studies on 11 published colorectal cancer risk loci

**DOI:** 10.1038/sj.bjc.6605774

**Published:** 2010-07-20

**Authors:** S von Holst, S Picelli, D Edler, C Lenander, J Dalén, F Hjern, N Lundqvist, U Lindforss, L Påhlman, K Smedh, A Törnqvist, J Holm, M Janson, M Andersson, S Ekelund, L Olsson, S Ghazi, N Papadogiannakis, A Tenesa, S M Farrington, H Campbell, M G Dunlop, A Lindblom

**Affiliations:** 1 Department of Molecular Medicine and Surgery, Karolinska Institutet, Stockholm S17176, Sweden; 2 Department of Clinical Sciences, Danderyd Hospital, Karolinska Institutet, Stockholm, Sweden; 3 Department of Surgery, St Görans Hospital, Stockholm, Sweden; 4 Division of Surgery, Department of Clinical Science, Karolinska Institutet at Danderyds Hospital, Stockholm, Sweden; 5 Norrtälje Hospital, Norrtälje, Sweden; 6 Department of Surgery, Uppsala University Hospital, Uppsala, Sweden; 7 Colorectal Unit, Department of Surgery, Central Hospital, Västerås, Sweden; 8 Department of Surgery, Central Hospital, Karlstad, Sweden; 9 Länssjukhuset Gävle-Sandviken, Gävle, Sweden; 10 Department of Clinical Science, Intervention and Techniques (CLINTEC), Karolinska Institutet, Karolinska University Hospital Huddinge, Sweden; 11 Department of Surgery, Örebro University Hospital, Örebro, Sweden; 12 Department of Clinical Science and Education, Södersjukhuset, Karolinska Institutet, Stockholm, Sweden; 13 Division of Pathology, Department of Laboratory Medicine, Karolinska Institute, Stockholm, Sweden; 14 Colon Cancer Genetics Group and Academic Coloproctology, Institute of Genetics and Molecular Medicine, University of Edinburgh, Edinburgh EH4 2XU, UK; 15 MRC Human Genetics Unit, Western General Hospital, Edinburgh EH4 2XU, UK; 16 Public Health Sciences, University of Edinburgh, Teviot Place, Edinburgh EH8 9AG, UK

**Keywords:** colorectal cancer, SNP (single-nucleotide polymorphism), association study, risk predisposition

## Abstract

**Background:**

Recently, several genome-wide association studies (GWAS) have independently found numerous loci at which common single-nucleotide polymorphisms (SNPs) modestly influence the risk of developing colorectal cancer. The aim of this study was to test 11 loci, reported to be associated with an increased or decreased risk of colorectal cancer: 8q23.3 (rs16892766), 8q24.21 (rs6983267), 9p24 (rs719725), 10p14 (rs10795668), 11q23.1 (rs3802842), 14q22.2 (rs4444235), 15q13.3 (rs4779584), 16q22.1 (rs9929218), 18q21.1 (rs4939827), 19q13.1 (rs10411210) and 20p12.3 (rs961253), in a Swedish-based cohort.

**Methods:**

The cohort was composed of 1786 cases and 1749 controls that were genotyped and analysed statistically. Genotype–phenotype analysis, for all 11 SNPs and sex, age of onset, family history of CRC and tumour location, was performed.

**Results:**

Of eleven loci, 5 showed statistically significant odds ratios similar to previously published findings: 8q23.3, 8q24.21, 10p14, 15q13.3 and 18q21.1. The remaining loci 11q23.1, 16q22.1, 19q13.1 and 20p12.3 showed weak trends but somehow similar to what was previously published. The loci 9p24 and 14q22.2 could not be confirmed. We show a higher number of risk alleles in affected individuals compared to controls. Four statistically significant genotype–phenotype associations were found; the G allele of rs6983267 was associated to older age, the G allele of rs1075668 was associated with a younger age and sporadic cases, and the T allele of rs10411210 was associated with younger age.

**Conclusions:**

Our study, using a Swedish population, supports most genetic variants published in GWAS. More studies are needed to validate the genotype–phenotype correlations.

Until some years ago, the candidate-gene approach was the only method available to the researchers for identifying potentially pathogenic variants. However, the fast technological development and the consequent acquisition of large amount of data in the past decade shifted the focus of research to genome-wide association studies (GWAS). Recent GWAS have identified multiple genetic loci associated with an increased or decreased risk of colorectal cancer (CRC) on 8q23.3, 8q24.21, 9p24, 10p14, 11q23.1, 14q22.2, 15q13.3, 16q22.1, 18q21.1, 19q13.1 and 20p12.3, explaining, at least to some extent, the genetics behind CRC as a complex disease (Broderick *et al*, 2007; Haiman *et al*, 2007; Tomlinson *et al*, 2007, 2008; Zanke *et al*, 2007; Houlston *et al*, 2008; Jaeger *et al*, 2008; Tenesa *et al*, 2008). Each of these loci is associated with a modest risk and, although fairly common they contribute very little to the overall burden of CRC. This case–control study focused on the known CRC single-nucleotide polymorphisms (SNPs) in a Swedish-based cohort and to compare our results with previous association studies in other populations. It also tested if there were more CRC patients than controls among individuals with higher number of risk alleles as reported previously (Tomlinson *et al*, 2010). Genotype–phenotype associations were analysed for age of onset, sex, family history of CRC and tumour location.

## Materials and methods

### Subjects

The case cohort was composed of 1786 consecutive CRC patients of Swedish origin recruited through the Swedish Low-Risk CRC Study Group from 14 different hospitals from central Sweden during 2004–2006. The mean age (at diagnosis) was 68.6 years (range 28–95 years), 53% were men and 47% were women and 22% had a family history of CRC among first- or second-degree relatives. The control cohort was composed of 1749 individuals as follows: 1319 blood donors from the general population between the age of 18 and 65 years and 430 unaffected spouses of CRC patients with the mean age of 66.3 (25–92) years, which were cancer-free and did not have a family history of any type of cancer.

### Loci and SNPs

Exploiting linkage disequilibrium between SNPs, we selected one SNP from each locus among those published. Thus we genotyped rs16892766 on 8q23.3, rs6983267 on 8q24.21, rs719725 on 9p24, rs10795668 on 10p14, rs3802842 on 11q23.1, rs4444235 on 14q22.2, rs4779584 on 15q13.3, rs9929218 on 16q22.1, rs4939827 on 18q21.1, rs10411210 on 19q13.1, rs961253 on 20p12.3 and excluded the following from the analysis: rs355527 on 20p12.3 (tagged by rs961253) and rs7259371 on 19q13.1 (tagged by rs10411210).

### Genotyping

Genomic DNA was extracted from peripheral blood by standard procedures. Six of the SNPs (rs9929218, rs719725, rs4444235, rs4779584, rs10411210 and rs961253) were genotyped using TaqMan SNP Genotyping Assay (Applied Biosystems, Foster City, CA, USA). Genotyping and first-quality check of the remaining five SNPs (rs6983267, rs16892766, rs10795668, rs4939827 and rs3802842) were performed, using a technology developed by Nanogen, at deCode Genetics, Reykjavik, Iceland (http://www.decode.com).

### Quality control

Sequencing was performed using Big-Dye terminator v3.1 cycle sequencing kit (Applied Biosystems), and fragments were separated on an ABI 3730 XL capillary sequencer. Chromatograms were analysed using SeqScape v2.5 (Applied Biosystems). Primers and amplification conditions are available upon request.

### Genotype–phenotype analysis

We studied sex, age of onset (early *vs* late, >60 years), family history of CRC (any case of CRC among first- or second-degree relatives), location, colon *vs* rectum and right *vs* left (proximal and distal to the splenic flexure).

### Statistical analysis

Deviations of the genotype frequencies in cases and controls from those expected under Hardy–Weinberg equilibrium were calculated by *χ*^2^-tests (one degree of freedom). Allelic frequencies of the SNPs in the case and control groups were compared using a *χ*^2^-test (allele 1 (common) *vs* allele 2 (minor)), except for rs6983267 where the common allele is suggested to be the risk allele (Tomlinson *et al*, 2007). To make comparisons, we chose to present risk and common allele according to previous publications. Analyses were also performed under various types of genetic models including the comparison of homozygotes (genotype 11 *vs* 22), the dominant (11 *vs* (12+22)), the recessive ((11+12) *vs* 22) models and the allele frequency difference ((1) *vs* (2)). In addition, Armitage's trend test, which takes into account the individuals’ genotypes rather than just alleles, (Sasieni, 1997) was performed using the DeFinetti programme provided as an online source (http://ihg2.helmholtz-muenchen.de/cgi-bin/hw/hwa1.pl). The significance level for statistical tests was set at 0.05. Odds ratios (ORs), their 95% confidence intervals (CIs) and their corresponding *P*-values were calculated using the same programme. The analyses were validated using Statistica 7.0 (StatSoft Inc., Tulsa, OK, USA). Statistical analysis for the clinical parameters was carried out with Statistica, using cross-tabulation analysis. Pearson *χ*^2^-test was used to calculate the *P*-value, and the level of significance was set at 0.05.

## Results

**Figure 1:**
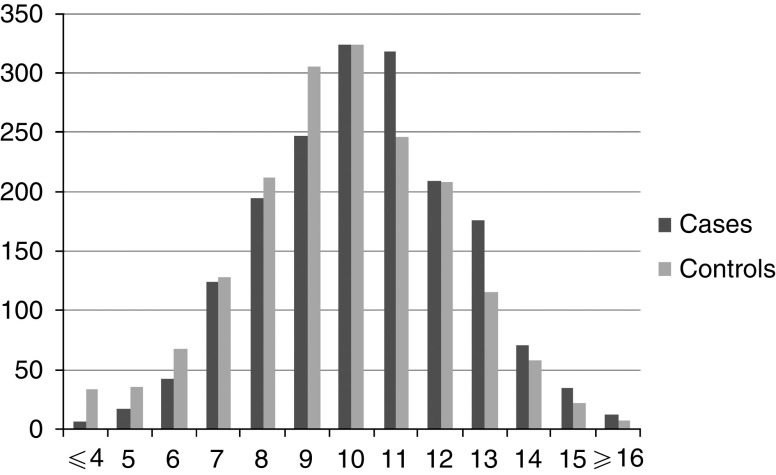
Polygenic model of 11 CRC-related SNPs. Distribution of risk alleles among cases and controls: black, cases; grey, controls.

Genotype frequencies of cases and controls as well as ORs and *P*-values for the different analyses are shown in Table 1. Significant associations between 5 of the 11 genotyped SNPs (rs16892766, rs6983267, rs10795668, rs4779584 and rs4939827) and CRC risk were confirmed and showed similar ORs as in previous publications (Broderick *et al*, 2007; Tomlinson *et al*, 2007, 2008; Jaeger *et al*, 2008). For SNP rs16892766 on 8q23.3, an increased risk of CRC was identified (*P*<0.002 for all analyses except the recessive model) with the highest OR equal to 1.34 (1.13–1.60) for the heterozygous. Likewise, the increased risk suggested for the variant rs6983267 on 8q24.21 was confirmed in all the analyses, with the highest OR equal to 1.37 (1.13–1.67) for the homozygous state. rs4779584 on 15q13.3 has been associated with an increased risk that could be confirmed for the heterozygous individuals, OR=1.18 (1.02–1.36). The protected effects suggested for rs10795668 on 10p14 and rs4939827 on 18q21.1 were both confirmed for homozygous and heterozygous with an OR equal to 0.66 (0.52–0.83) and OR 0.82 (0.70–0.96), respectively. The ORs for rs3802842 on 11q23.1 showed a trend with an OR equal to 1.27 (NS) for homozygous. The rs9929218 on 16q22.1, rs10411210 on 19q13.1 and rs961253 on 20p12.3 showed weak trends in the same direction as published (NS), whereas the two SNPs rs719725 on 9p24 and rs4444235 on 14q22.2 were not confirmed. The distribution of risk alleles between cases and controls in the Swedish population is shown in Figure 1. There is a clear shift with a higher number of alleles in affected individuals compared to controls.

Genotype–phenotype analysis was performed for all 11 loci and for sex, age, family history and tumour location, and the *P*-values for all analyses are shown in Table 2. Four associations were found, three for age and one for family history (Table 3). Being homozygous for the risk allele G for rs6983267 showed association to older age (*P*=0.0014). In contrast, for rs1075668 the risk allele G was associated with younger age (*P*=0.035) and sporadic cases (*P*=0.047). The T allele of rs10411210 was associated with younger age (*P*=0.045) in homozygotes (Table 3).

## Discussion

We studied SNPs on 11 loci published to be associated with an increased or decreased risk for CRC and were able to show statistically significant results for 5 of them. The first SNP, rs6983267 on 8q24.21, was published by Tomlinson *et al* (2007), where the most common allele G was suggested to be the risk allele. Our study showed similar results as previous studies in other populations (Berndt *et al*, 2008; Tuupanen *et al*, 2008; Wokolorczyk *et al*, 2008; Curtin *et al*, 2009; Middeldorp *et al*, 2009). Likewise, the SNP rs16892766 on 8q23.3 was similar to both the GWAS study and one replicative study (Tomlinson *et al*, 2008; Wijnen *et al*, 2009). The protective effect associated with rs10795668 on 10p14 was confirmed for homozygous carriers in the Swedish material (Tomlinson *et al*, 2008). The SNP rs4779584 on 15q13.3, published by Jaeger *et al* (2008) as a risk association with CRC was confirmed by us. For the SNP rs4939827 on 18q21.1, Broderick *et al* published the variant to be protective, which could also be shown by us and one previous study (Curtin *et al*, 2009). The SNP rs3802842 on 11q23.1 was first published by Tenesa and co-workers and confirmed by others (Pittman *et al*, 2008; Middeldorp *et al*, 2009; Wijnen *et al*, 2009). Our results were similar, but not statistically significant. This discrepancy could be due to different populations, sample size or study design. Wijnen *et al* (2009) used a Dutch population, and used mismatch repair gene carriers only and no controls. The majority of the Dutch samples (995 cases and 1340 controls) used by Middeldorp *et al* (2009) were familial cases and Pittman *et al* (2008) used eight independent case–control series (10 638 cases and 10 457 controls) and were able to confirm significant values for most of the populations.

No association was detected for rs719725 on 9p24, initially reported in cohorts from Canada, the United States, Newfoundland, Scotland and France, which the authors themselves were unable to replicate in a second French cohort (Zanke *et al*, 2007). Later it was confirmed in cohorts from the American, Canadian and Australian populations (Poynter *et al*, 2007). Even though the distribution of the three genotypes was the same, we hypothesise that this negative result could be due to its population specificity and the causal SNP being on different haplotypes or were these results false positives. A study using British and American cohorts was also unable to detect any association for this SNP (Curtin *et al*, 2009).

To our knowledge, none of the remaining four SNPs has been studied in other populations yet. In fact, the confirmed five loci were the first ones to be published whereas the SNPs on 14q22.2, 16q22.1, 19q13.1 and 20p12.3 were only captured by meta-analysis of large GWAS (Houlston *et al*, 2008), suggesting that these four could be more difficult to replicate in follow-up studies. The three SNPs on 16q22.1, 19q13.1 and 20p12.3 did not show statistically significant values in our study. However, when looking at the ORs in the Swedish samples, association was suggested but with a wider CI compared to the first report (Houlston *et al*, 2008). Finally, we were unable to confirm association to CRC risk for rs4444235 on 14q22.2 (Houlston *et al*, 2008), which again could be due to a smaller size or possibly a population difference.

Another possible explanation for the different results could depend on different genotype frequencies among populations or methods used for genotyping. For all SNPs the genotype frequencies in Swedish samples were similar to published data. Regarding methods, SNP arrays were used for the GWAS, whereas other studies used Sequenom's iPLEX Gold (San Diego, CA, USA), genomic sequencing, SNPlex, PCR KASPar or TaqMan. This does not immediately explain the different results in the Swedish material. Because four of the five SNPs genotyped by DeCode and rs4778495 genotyped in Edinburgh using TaqMan were confirmed, while none of the five (rs9929218, rs719725, rs4444235, rs10411210 and rs961253) carried out in our lab showed statistically significant results we validated the results from our TaqMan analysis. In total 1000 cases and 1000 controls were sequenced for the five SNPs. The concordance was 99.8%, why we do not think that the method explains the difference between our study results and previous publications.

Carrying one risk variant alone is neither necessary nor sufficient for developing CRC. However, in Figure 1 we show support for the general idea that the CRC patients carry more risk alleles compared to controls. For both cases and controls, the distribution is outlined in the diagram of carriers with a shift toward higher numbers of risk alleles in affected individuals, in line with what has been published (Tomlinson *et al*, 2010). Even though we did not confirm all SNPs, and used 11 SNPs instead of 10, the distribution of risk alleles showed very similar data (Figure 1) to what was published that further strengthens the results and confirms the genetic contribution by the alleles overall (Tomlinson *et al*, 2010).

The genotype–phenotype analysis interestingly showed four associations for three SNPs. Other studies have published genotype–phenotype analysis for only one of the loci, 8q24.21, and sex, tumour site, age at diagnosis and family history (Haiman *et al*, 2007; Poynter *et al*, 2007; Tuupanen *et al*, 2008). We report an association to age for rs6983267 on 8q24.21; the risk allele G was associated to our older patients (*P*=0.0014). This was not seen in any of the other studies (Haiman *et al*, 2007; Poynter *et al*, 2007; Tuupanen *et al*, 2008), perhaps because of the different age groups used. In contrast to our study and the two other studies, Tuupanen *et al* (2008) for the same SNP reported an association to family history. This is not likely to depend on the definition of family history, because only our study used a different classification from the other three. In line with our results, no one found any support for sex or tumour site (Haiman *et al*, 2007; Poynter *et al*, 2007; Tuupanen *et al*, 2008). For rs10795668 on 10p14, we showed association to age and family history. Being homozygous for the risk alleles was associated to younger patients (*P*=0.035) and to sporadic cases (*P*=0.047). For rs10411210 an association was identified for being homozygous for the risk allele in younger patients (*P*=0.045). Replications of these genotype–phenotype analyses are needed before any conclusion can be made.

The genetic contribution to CRC as a whole has been estimated to be as high as 35% (Lichtenstein *et al*, 2000). Although very common in the general population, considering an additive model of inheritance the 10 SNPs discovered so far (9p24 excluded) account jointly for only about 6% of the excess genetic risk (Houlston *et al*, 2008). These statements leave the majority of the genetic contribution to CRC development still unexplained and more studies aiming to define additional SNPs and hopefully also some more high-penetrant predisposing genes are welcomed.

## Figures and Tables

**Table 1:** OR for case–control study of 11 published CRC susceptibility loci

**Locus/SNP**	**OR published**	**Genotypes**	**No cases (%)**	**No controls (%)**	**OR (95% CI)**	***P*-values**
8q23.3		AA	1379 (79)	1404(83)	1	
rs16892766	1.27 (het)	AC	356 (20)	270 (16)	**1.34 (1.13–1.60)**	**0.0009**
	1.43 (hom)	CC	20 (1)	17 (1)	1.20 (0.63–2.30)	0.586
	Tomlinson *et al*, 2008	AC+CC			**1.33 (1.13–1.58)**	**0.0009**
		Allelic			**1.29 (1.10–1.51)**	**0.0016**
		Trend			**1.26**	**0.0017**
						
8q24.21		TT	397 (23)	332 (19)	1	
rs6983267	1.27 (het)	TG	890 (51)	892 (51)	**1.20 (1.01–1.43)**	**0.04**
	1.47 (hom)	GG	450 (26)	517 (30)	**1.37 (1.13–1.67)**	**0.001**
	Tomlinson *et al*, 2007	AG+GG			**1.26 (1.07–1.48)**	**0.006**
		Allelic			**1.16 (1.06–1.28)**	**0.0015**
		Trend			**1.17**	**0.001**
						
9p24	1.14 (com)	AA	672 (39)	669 (39)	1	
rs719725		AC	821 (48)	797 (46)	1.03 (0.89–1.19)	0.733
	Zanke *et al*, 2007	CC	231 (13)	253 (15)	0.91 (0.74–1.12)	0.368
		AC+CC			0.997(0.87–1.14)	0.971
		Allelic			0.97 (0.88–1.07)	0.554
		Trend			0.96	0.554
						
10p14		GG	853 (48)	745 (44)	1	
rs10795668	0.87 (het)	GA	779 (44)	754 (44)	0.90 (0.78–1.04)	0.151
	0.80 (hom)	AA	148 (8)	197 (12)	**0.66 (0.52–0.83)**	**0.0004**
	Tomlinson *et al*, 2008	GA+AA			**0.85 (0.75–0.97)**	**0.018**
		Allelic			**0.85 (0.76–0.94)**	**0.001**
		Trend			**0.83**	**0.001**
						
11q23.1	1.11 (com)	AA	941 (53)	926 (55)	1	
rs3802842		AC	688 (39)	656 (39)	1.03 (0.90–1.19)	0.659
	Tenesa *et al*, 2008	CC	142 (8)	110 (6)	1.27 (0.98–1.66)	0.076
		AC+CC			1.07 (0.93–1.22)	0.347
		Allelic			1.08 (0.97–1.21)	0.143
		Trend			1.1	0.145
						
14q22.2		TT	573 (33)	533 (32)	1	
rs4444235	1.13 (het)	TC	829 (47)	838 (49)	0.92 (0.79–1.07)	0.284
	1.23 (hom)	CC	356 (20)	326 (19)	1.02 (0.84–1.23)	0.872
	Houlston *et al*, 2008	TC+CC			0.95 (0.82–1.09)	0.455
		Allelic			0.997(0.91–1.10)	0.951
		Trend			1.00	0.952
						
15q13.3		CC	1050 (61)	1104 (65)	1	
rs4779584	1.23 (het)	CT	572 (33)	511 (30)	**1.18 (1.02–1.36)**	**0.029**
	1.70 (hom)	TT	94 (6)	89 (5)	1.11 (0.82–1.50)	0.496
	Jaeger *et al*, 2008	CT+TT			**1.17 (1.02–1.34)**	**0.029**
		Allelic			1.12 (1.00–1.26)	0.051
		Trend			1.096	0.057
						
16q22.1		GG	929 (53)	913 (54)	1	
rs9929218	0.92 (het)	GA	700 (40)	648 (38)	1.06 (0.92–1.22)	0.404
	0.82 (hom)	AA	113 (7)	138 (8)	0.81 (0.62–1.05)	0.108
	Houlston *et al*, 2008	GA+AA			1.02 (0.90–1.16)	0.810
		Allelic			1.12 (1.00–1.26)	0.051
		Trend			0.945	0.566
						
18q21.1		TT	501 (28)	408 (24)	1	
rs4939827	0.86 (het)	TC	886 (50)	884 (53)	**0.82 (0.70–0.96)**	**0.013**
	0.73 (hom)	CC	395 (22)	387 (23)	0.83 (0.69–1.01)	0.059
	Broderick *et al*, 2007	TC+CC			**0.82 (0.71–0.96)**	**0.011**
		Allelic			0.91 (0.83–1.00)	0.051
		Trend			**0.91**	**0.048**
						
19q13.1		CC	1490 (84)	1421(83)	1	
rs10411210	0.87 (het)	CT	264 (15)	272 (16)	0.93 (0.77–1.11)	0.411
	0.72 (hom)	TT	13 (1)	14 (1)	0.89 (0.42–1.89)	0.753
	Houlston *et al*, 2008	CT+TT			0.92 (0.77–1.11)	0.389
		Allelic			0.93 (0.78–1.10)	0.385
		Trend			0.930	0.387
						
20p12.3		CC	694 (39)	693 (40)	1	
rs961253	1.14 (het)	CA	806 (46)	791 (46)	1.02 (0.88–1.18)	0.813
	1.24 (hom)	AA	265 (15)	237 (14)	1.12 (0.91–1.37)	0.290
	Houlston *et al*, 2008	CA+AA			1.04 (0.91–1.19)	0.568
		Allelic			1.05 (0.95–1.16)	0.344
		Trend			1.05	0.349

Abbreviations: allelic=allele frequency difference; trend=Armitage's trend test; com=common odds ratio; hom=homozygous; het=heterozygous; all=allelic.

Minor allele frequencies Swedish cohort cases/controls: 8q23.3 (0.11/0.09), 8q24.21 (0.49/0.45), 9p24 (0.37/0.38), 10p14 (0.30/0.34), 11q23.1 (0.27/0.26), 14q22.2 (0.44/0.44), 15q13.3 (0.22/0.20), 16q22.1 (0.27/0.27), 18q21.1 (0.47/0.49), 19q13.1 (0.08/0.09), 20p12.3 (0.38/0.37). The bold values indicate *P*<0.05.

**Table 2:** *P*-values from genotype–phenotype analysis of 11 CRC susceptibility loci

**SNP/loci**	**rs16892766**	**rs6983267**	**rs719725**	**rs10795668**	**rs3802842**	**rs4444235**	**rs4779584**	**rs9929218**	**rs4939827**	**rs10411210**	**rs961253**
**Phenotype**	**8q23.3**	**8q24.21**	**9p24**	**10p14**	**11q23.1**	**14q22.2**	**15q13.3**	**16q22.1**	**18q21.1**	**19q13.1**	**20p12.3**
Age of onset	*P*=0.277	***P*=0.0014**	*P*=0.414	***P*=0.035**	*P*=0.683	*P*=0.070	*P*=0.782	*P*=0.055	*P*=0.606	***P*=0.045**	*P*=0.853
Sex	*P*=0.251	*P*=0.190	*P*=0.547	*P*=0.313	*P*=0.892	*P*=0.880	*P*=0.688	*P*=0.784	*P*=0.569	*P*=0.069	*P*=0.561
Family history	*P*=0.998	*P*=0.131	*P*=0.474	***P*=0.046**	*P*=0.607	*P*=0.587	*P*=0.861	*P*=0.459	*P*=0.131	*P*=0.071	*P*=0.489
Rectum/colon	*P*=1.000	*P*=0.789	*P*=0.705	*P*=0.170	*P*=0.787	*P*=0.407	*P*=0.229	*P*=0.184	*P*=0.472	*P*=0.632	*P*=0.159
Location left/right	*P*=0.469	*P*=0.277	*P*=0.240	*P*=0.246	*P*=0.962	*P*=0.347	*P*=0.309	*P*=0.709	*P*=0.314	*P*=0.505	*P*=0.985

Genotype–phenotype analysis. The bold values indicate *P*<0.05.

**Table 3:** Genotype–phenotype analysis for the four analysis with statistically significant results

	**Age**		
**rs6983267 8q24.21**	**⩽60% (no.)**	**>60% (no.)**	**Total number of cases**
TT	22.7 (88)	18.0 (241)	
GT	54.6 (212)	50.2 (674)	
GG	22.7 (88)	31.8 (427)	
*P*=0.0014			1730
	**Age**		
**rs10795668 10p14**	**⩽60% (no.)**	**>60% (no.)**	**Total number of cases**
AA	8.6 (34)	8.2 (113)	
AG	38.1 (151)	45.3 (622)	
GG	53.3 (211)	46.5 (637)	
*P*=0.035			1768
	**Family history**		
**rs10795668 10p14**	**Sporadic % (no.)**	**Familial % (no.)**	**Total number of cases**
AA	7.5 (101)	10.8 (42)	
AG	43.1 (581)	45.1 (175)	
GG	49.4 (667)	44.1 (171)	
*P*=0.046			1737
	**Age**		
**rs10411210 19q13.1**	**⩽60% (no.)**	**>60% (no.)**	**Total number of cases**
TT	1.5 (6)	0.5 (7)	
CT	12.7 (50)	15.7 (214)	
CC	85.8 (339)	83.8 (1144)	
*P*=0.045			1760
